# Changes in Greenhouse Grown Tomatoes Metabolite Content Depending on Supplemental Light Quality

**DOI:** 10.3389/fnut.2022.830186

**Published:** 2022-03-22

**Authors:** Ina Alsina, Ieva Erdberga, Mara Duma, Reinis Alksnis, Laila Dubova

**Affiliations:** ^1^Faculty of Agriculture, Institute of Soil and Plant Sciences, Latvia University of Life Sciences and Technologies, Jelgava, Latvia; ^2^Department of Chemistry, Faculty of Food Technology, Latvia University of Life Sciences and Technologies, Jelgava, Latvia; ^3^Department of Mathematics, Faculty of Information Technologies, Latvia University of Life Sciences and Technologies, Jelgava, Latvia

**Keywords:** tomatoes, LED, HPSL, lycopene, taste index, phenols, flavonoids

## Abstract

Tomatoes (*Solanum lycopersicum* L.) are good source of several biologically active compounds and antioxidants, especially lycopene, phenolic compounds, and vitamins. Tomatoes are found all over the world and are cultivated in a wide variety of environmental conditions. Light-emitting diode (LED) lamps are increasingly being used in the cultivation of tomatoes due to their cost-effectiveness and wide range of possibilities to adapt the spectrum of light emitted to the needs of plants. The aim of this study is to evaluate the effect of different additional lighting used in the greenhouse on the accumulation of biologically active compounds in different varieties of tomato fruit. Chemical composition—content of organic acids, lycopene, total carotenoids, total phenolics and flavonoids as well as dry matter, soluble solids content, and taste index were determined in five tomato cultivars (“Bolzano F1,” “Chocomate F1,” “Diamont F1,” “Encore F1,” and “Strabena F1”), which were cultivated in greenhouse in an autumn-spring season by using additional lighting with 16 h photoperiod. Three different lighting sources were used: LED, induction (IND) lamp, and high-pressure sodium lamp (HPSL). Experiments were performed during 3 years. Results showed that tomato varieties react differently to the supplemental lighting used. Cultivars, such as “Encore” and “Strabena,” are the most unresponsive to supplemental light. Experiments have shown that HPSL stimulates the accumulation of primary metabolites in tomato fruit. In all the cases, soluble solids content was 4.7–18.2% higher as compared to other lighting sources. As LED and IND lamps emit about 20% blue-violet light, the results suggest that blue-violet light of the spectrum stimulates the accumulation of phenolic compounds in the fruit by 1.6–47.4% under IND and 10.2–15.6% under LED compared to HPSL. Red fruit varieties tend to synthesize more β-carotene under supplemental LED and IND light. An increase of blue promotes the synthesis of secondary metabolites.

## Introduction

As understanding of the importance of diet in ensuring quality and sustainability of human life grows, the pressure on the agricultural sector as a basic element in securing food quality is increasing. Tomatoes, as the second most grown vegetable [according to the Food and Agriculture Organization (FAO) statistics for 2019], are an important part of the cuisine of almost every nation. The limited caloric supply, relatively high fiber content, and presence of mineral elements, vitamins, and phenols, such as flavonoids, make the tomato fruit an excellent “functional food” providing many physiological benefits and basic nutritional requirements (1). The biochemically active substances found in tomatoes, mainly due to their high antioxidant capacity, are recognized not only for the general improvement of health, but also as a therapeutic option against various diseases, such as diabetes, heart diseases, and toxicities (2–4). Ripe tomato fruit contains an average 3.0–8.88% dry matter, which consists of 25% fructose, 22% glucose, 1% sucrose, 9% citric acid, 4% malic acid, 8% mineral elements, 8% protein, 7% pectin, 6% cellulose, 4% hemicellulose, 2% lipids, and the remaining 4% are amino acids, vitamins, phenolic compounds, and pigments (5, 6). The composition of these compounds varies depending on genotype, growing conditions, and fruit development stage. Tomato plants are highly sensitive to environmental factors, such as light conditions, temperature, and the amount of water in the substrate, which lead to changes in plant metabolism, which, in turn, affect the quality and chemical composition of the fruit (7). Environmental conditions affect both the tomato physiology and the synthesis of secondary metabolites. Plants grown under stress conditions react by increasing their antioxidant properties (8).

The origin of tomatoes as a species is linked to the Central American region (9) and techniques, such as the construction of greenhouses to supply the necessary temperature and light for tomatoes, are often required to provide the necessary agroclimatic conditions, especially in the temperate climatic zone and during the winter season. Under such conditions, light is often the limiting factor for tomato development. Supplementary lighting during winter and early spring seasons allows producing high-quality tomatoes during the low solar irradiance period (10). The use of lamps with different wavelengths cannot only ensure a sufficient tomato yield, but also change the biochemical composition of tomato fruit. For the last 60 years, high-pressure sodium lamps (HPSLs) have been used in the greenhouse industry due to their long operating life and low acquisition costs (11). However, in the last years, light-emitting diodes (LEDs) have become increasingly popular as a more energy-saving alternative (12). Supplemental LED has been used as an efficient light source to meet the demand for tomatoes production. Lycopene and lutein contents in tomatoes were 18 and 142% higher when they were exposed to the supplemental LED lighting. However, β-carotene content did not differ between the light treatments (12). LED blue and red light increased lycopene and β-carotene content (13), resulting in the early ripening of tomato fruit (14). Soluble sugar contents of the ripe tomato fruit were decreased by longer far-red (FR) light durations (15). Analogous conclusions were drawn in the study by Xie: red light induces lycopene accumulation, but FR light reverses this effect (13). There is less information on the effects of blue light on tomato fruit development, but studies show that blue light has a lesser effect on the amount of biochemical compounds in tomato fruit, but more on process stability. For example, Kong and others have found that blue light is better used to prolong the shelf-life of tomatoes, as blue light significantly increases the firmness of the fruit (16), which essentially means that blue light slows down the ripening process, which leads to an increase in amount of sugars and pigments. The use of greenhouse coverings as a means of regulating the composition of light proves a similar pattern. The use of a coating with a higher red and lower blue light transmission increases the lycopene content by about 25%. In combination with a photoperiod increased from 11 to 12 h, the amount of lycopene increases by about 70% (17). It is not always possible in studies to accurately distinguish the effect of factors on changes in the chemical composition of tomato fruit. Especially, in greenhouse conditions, the composition of the fruit can be increased by elevated temperatures or reduced water levels. In addition, these factors may correlate with the genotype-specific to the variety and development stage (1, 18). Water deficit may benefit tomatofruit quality due to increased levels of total soluble solids (sugars, amino acids, and organic acids), which are major compounds accumulated in fruit. A rise of soluble solids improves the quality of fruits because it affects the flavor and taste (8).

Despite the reported effects of light spectrum on the accumulation of plant metabolites, the wider knowledge of different spectrum effects for improving the quality of tomatoes is required. Accordingly, the aim of this study is to evaluate the effect of additional lighting used in the greenhouse on the accumulation of primary and secondary metabolites in different tomatoes varieties. Changes in the spectral content of lighting system can alter the composition of primary and secondary metabolites in tomato fruit. The acquired knowledge will improve the understanding of the effect of light on the relationship between yield and its quality.

## Materials and Methods

### Plant Material and Growing Conditions

Experiments were conducted in greenhouse (4 mm cell polycarbonate) of the Institute of Soil and Plant Sciences, Latvia University of Life Sciences and Technologies 56°39'N 23°43'E during 2018/2019, 2019/2020, and 2020/2021 late autumn-early spring seasons.

Commercially grafted tomato (*Solanum lycopersicum* L.) cultivars “Bolzano F1” (fruit color—orange), “Chocomate F1” (fruit color—red-brown), and red fruit cultivars “Diamont F1,” “Encore F1,” and “Strabena F1” were used. Each plant had two leading heads and during growth, it was trellised on a high–wire system. Obtained plants, first, were transplanted in black 5 L plastic containers with “Laflora” peat substrate KKS-2, pH_KCl_ 5.2–6.0, and fraction size 0–20 mm, PG mixture (NPK 15-10-20) 1.2 kg m^−3^, Ca 1.78%, and Mg 0.21%. When plants reached anthesis, they were transplanted into 15 L black plastic containers with the same “Laflora” peat substrate KKS-2. Plants were fertilized once a week with 1% solution of Kristalon Green (NPK 18-18-18) with Mg, S, and microelements during the vegetative phase of plant growth and with Kristalon Red (NPK 12-12-36) with microelements or 1% Ca(NO_3_)_2_ during the reproductive phase, in proportion 300 ml per L of substratum.

The water content in the vegetation containers was maintained at 50–80% of the full water holding capacity. Average day/night temperatures were 20–22°C/17–18°C. Maximal temperature during the day (March) did not exceed 32°C and minimal temperature (November) during the night was not <12°C. Temperature has also been measured under the lamps at the distance 50, 100, and 150 cm from the luminaire. It was detected that under the HPSL 50 cm from the luminaire, temperature was 1.5°C higher than under the others. Temperature differences at the fruit level were not detected.

### Lighting Conditions

**Figure 1:**
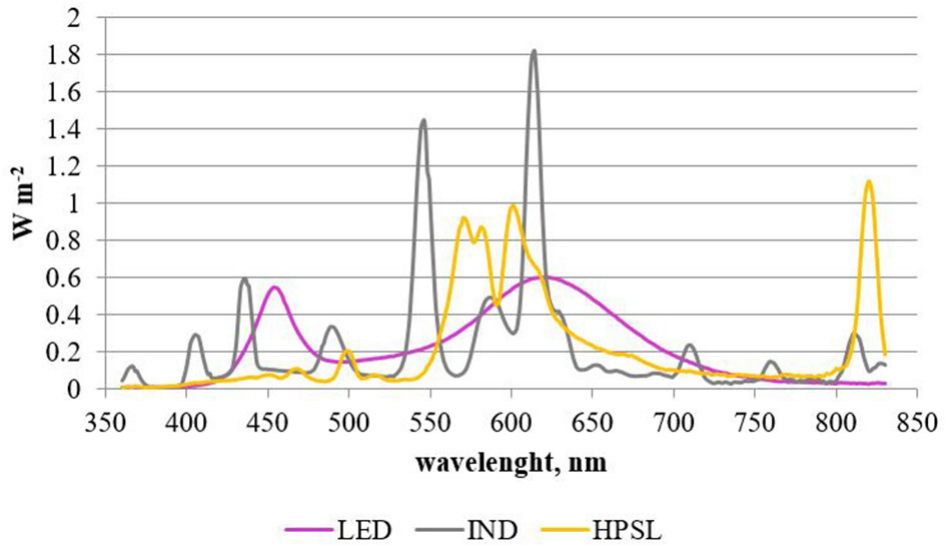
Spectrum of lighting sources.

**Figure 2:**
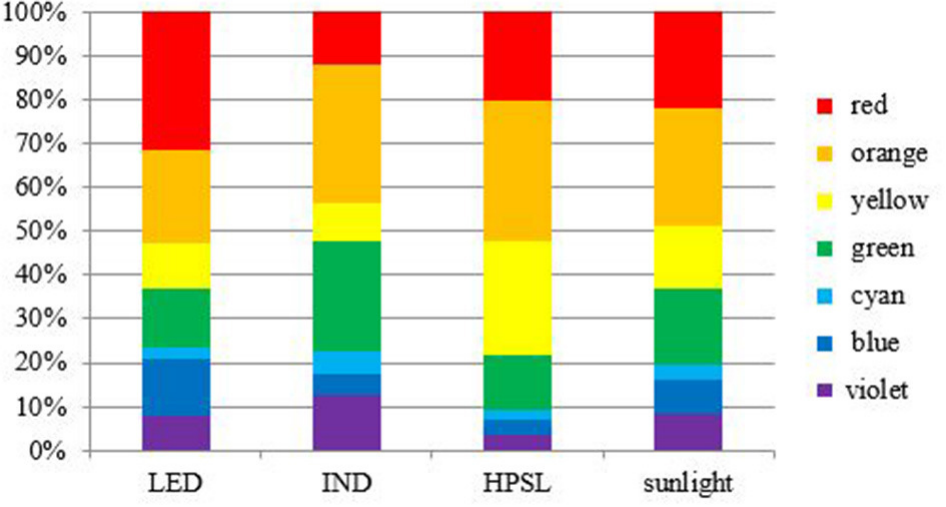
Spectral distribution of lighting source.

Tomatoes were cultivated in autumn-spring seasons by using additional lighting with a 16 h photoperiod. Three different lighting sources were used: Led cob Helle top LED 280 (LED), induction (IND) lamp, and HPSL Helle Magna (HPSL). At the apex height, plants received 200 ± 30 μmol m^−2^ s^−1^ under LED and HPSL and 170 ± 30 μmol m^−2^ s^−1^ under IND lamps. Distribution of light radiance is shown in Figures 1, 2. Light intensity and spectral distribution were detected by handheld spectral light meter MSC15 (Gigahertz Optik GmbH, Türkenfeld, Germany, UK).

The used lamps differed in their light spectral distribution. The most similar to sunlight in the red part (625–700 nm) of the spectrum was HPSL. The IND lamp in this part of the spectrum gave 23.5% less light, but LED was close to 2 times more. Orange light (590–625 nm) was emitted mostly by HPSL, green light (500–565 nm) was emitted mostly by IND, blue light (450–485 nm) was emitted mostly by LED, but purple light (380–450 nm) was emitted mostly by IND lamp. When comparing the whole spectrum of visible light, the LED light source should be considered as the closest to sunlight and the IND should be considered as the most inappropriate in terms of spectrum.

### Extraction and Determination of Phytochemicals

Tomato fruits were harvested on the full ripeness stage. Fruits were harvested once a month starting in the middle of November and ending in March. All the fruits were counted and weighted. At least, 5 fruits from each variant (for cv “Strabena” −8–10 fruits) were sampled for analyses. Tomato fruits were ground into a puree by using a hand blender. For each evaluated parameter, three replications were analyzed.

#### Determination of Lycopene and β-Carotene

To determine the concentration of lycopene and β-carotene, a sample of 0.5 ± 0.001 g from the tomato puree was then weighed into a tube and 10 mL of tetrahydrofuran (THF) was added (19). The tubes were sealed and kept at room temperature for 15 min, shaking occasionally, and finally centrifuged for 10 min at 5,000 rpm. The absorbance of the supernatants obtained was determined spectrophotometrically by measuring the absorbance at 663, 645, 505, and 453 nm and then the lycopene and β-carotene contents (mg 100 mL^−1^) were calculated according to the following equation.


(1) $$\begin{array}{l} C_{lyc}=-0.0458\times A_{663}+0.204\times A_{645}+0.372\times A_{505}\\ \;-0.0806\times A_{453} \end{array}$$

(2) $$\begin{array}{l} C_{car}=0.216\times A_{663}-1.22\times A_{645}-0.304\times A_{505}\\ \;+0.452\times A_{453} \end{array}$$

where A663, A645, A505, and A453—absorption at corresponding wavelength (20).

The lycopene and β-carotene concentrations are expressed as mg ${\text{g}}_{\text{FM}}^{-1}$.

#### Determination of Total Phenols

A sample of 1 ± 0.001 g from the tomato puree was weighed into a graduated tube and 10 ml of solvent (methanol/distilled water/hydrochloric acid 79:20:1) was added. The graduated tubes were sealed and shaken for 60 min at 20°C in the dark and then centrifuged for 10 min at 5,000 rpm. The total phenol concentration was determined by using the Folin–Ciocalteu spectrophotometric method (21) with some modifications: Folin–Ciocalteu reagent (diluted 10-fold in distilled water) was added to 0.5 ml of the extract and after 3 min add 2 mL of sodium carbonate (Na_2_CO_3_) (75 gL^−1^). The sample was mixed and after 2 h incubation at room temperature in the dark, the absorbance at 760 nm was measured. The concentration of total phenolic compounds was calculated by using the calibration curve and obtained equation 3, and expressed as gallic acid equivalent (GAE) per 100 g of fresh tomato mass.


(3) $$\begin{array}{l} Phe=\frac{0.556\times \left(A_{760}+0.09\right)\times 100}{m} \end{array}$$

where A_760_–absorption at corresponding wavelength and m—mass of the sample.

#### Determination of Flavonoids

A sample of 1 ± 0.001 g from the tomato puree was weighed into a graduated tube and 10 mL ethanol was added. The graduated tubes were sealed and shaken for 60 min at 20°C in the dark and then centrifuged for 10 min at 5,000 rpm. The colorimetric method (22) was used to determine flavonoids with minor changes: 2 mL of distilled water and 0.15 mL of 5% sodium nitrite (NaNO_2_) solution were added to 0.5 mL of the extract. After 5 min, a 0.15-mL of 10% solution of aluminum chloride (AlCl_3_) was added. The mixture was allowed to stand for another 5 min and 1 mL 1 M sodium hydroxide (NaOH) solution was added. The sample was mixed and after 15 min at room temperature, the absorbance at 415 nm was measured. The total flavonoid concentration was calculated by using calibration curve and Equation 4 and expressed as the amount of catechin equivalents (CEs) per 100 g of fresh tomato weight.


(4) $$\begin{array}{l} Fla=\frac{0.444\times A_{415}\times 100}{m} \end{array}$$

where A_415_–absorption at corresponding wavelength and m—mass of the sample.

#### Determination of Dry Matter and Soluble Solids

Dry matter was determined by drying samples in the thermostat at 60°C.

The total soluble solids content (expressed as °Brix) was measured with a refractometer (A.KRÜSS Optronic Digital Handheld Refractometer Dr301-95) calibrated at 20°C with distilled water.

#### Determination of Titratable Acidity (TA)

A sample of 2 ± 0.01 g from the tomato puree was weighed into a graduated tube and distilled water was added till 20 mL. The graduated tubes were sealed and shaken for 60 min at room temperature and then centrifuged for 10 min at 5,000 rpm. 5 mL aliquots were titrated with 0.1 M NaOH in the presence of phenolphthalein.


(5) $$\begin{array}{l} TA=\frac{V_{NaOH}\times V_{t}}{V_{s}\times m} \end{array}$$

where V_NaOH_–volume of used 0.1 M NaOH, Vt—total volume (20 mL), and Vs—sampled volume (5 mL).

Results are expressed as mg of citric acid per 100 g of fresh tomato weight. 1 mL 0.1 M NaOH corresponds to 6.4 mg citric acid.

#### Determination of Taste Index (TI)

A TI was calculated by using equation 6 (23).


(6) $$TI=\;\frac{{}^{\circ}\text{Brix}}{\text{20}\times TA}\text{+}TA$$

### Statistical Analyses

The normality and homogeneity of the descriptive statistics were tested for 354 observations. The Shapiro–Wilk test was used for the evaluation of normality within each combination of variety and lighting treatment. To estimate homogeneity of variances, Levene's test was conducted. The Kruskal–Wallis test was used to examine the differences between lighting conditions. When statistically significant differences were identified, the Wilcoxon *post-hoc* test with Bonferroni corrections was used for pairwise comparisons. The significance level used in the text, tables and graphs is α = 5%, unless stated otherwise.

## Results

Tomato fruit size and fruit biochemical parameters are genetically determined parameters, but cultivation conditions have a significant impact on these features. The largest fruits are harvested from “Diamont” (88.3 ± 22.9 g) and the smallest fruits are harvested from “Strabena” (13.0 ± 3.8 g), which are a variety of cherry tomatoes. The size of the fruit within the variety also varied from the time of harvest. The largest fruits were harvested at the beginning of production and the size of the tomatoes decreased as the plants grew. However, it should be noted that with the increased proportion of natural light at the end of March, tomatoes size slightly increased.

In all three years, the highest tomato yield was harvested using HPSL as additional lighting. The yield decrease under LED's was 16.0%, and under IND - 17.7% compared with HPSL. Different varieties of tomatoes reacted differently to supplemental lighting. Yield increase, although statistically insignificant, were observed for the cv “Strabena”, “Chocomate” and “Diamont” under LEDs. For cv “Bolzano” neither LED nor IND additional lighting was suitable, the reduction of total yield by 25–31% was observed.

**Figure 3:**
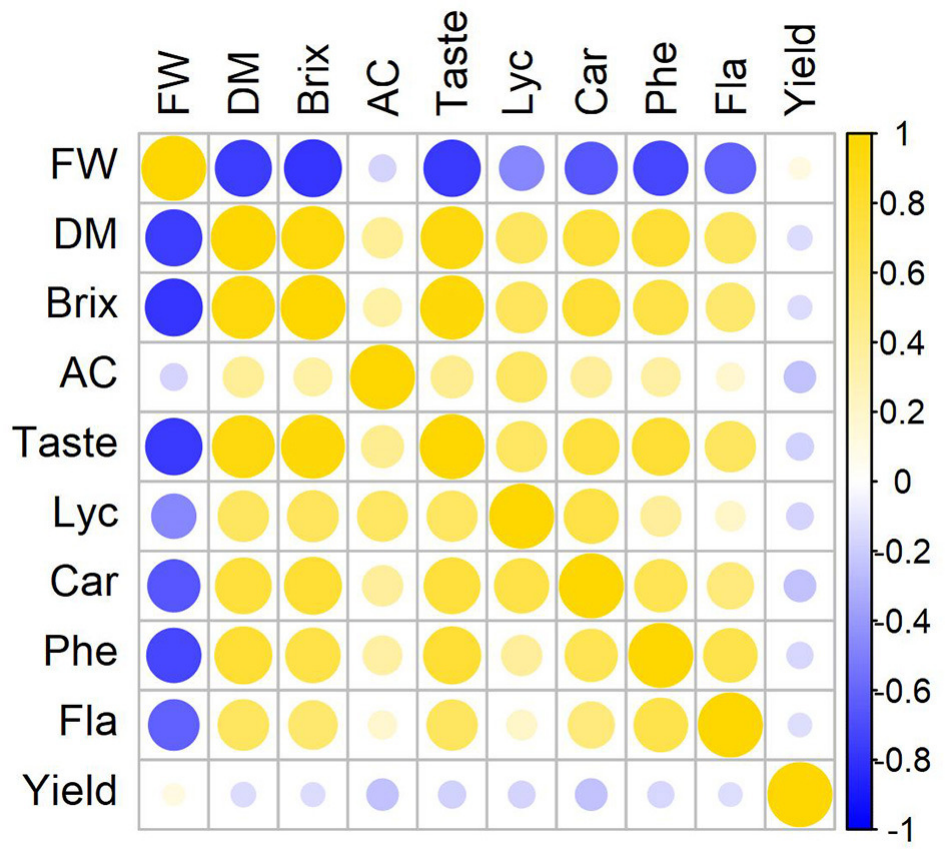
Correlations between the fruit parameters of tomatoes.

In average, larger tomato fruits contain less dry matter and soluble solids, they are not so tasty, and contain less carotenoids and phenols. The factor that is least affected by fruit size is the acid content. A high correlation is observed between the dry matter and soluble solids content and the TI (*r*_*n* = 195_ > 0.9). The correlation coefficient between the dry matter or soluble solids content and the carotenoid (lycopene and carotene) and the phenol content ranges between 0.7 and 0.8 (Figure 3).

Experiments have shown that, although the differences in the studied parameters between the lights used are sometimes large, there are few such parameters that would change significantly under the influence of the light source used during the whole growing season and taking into account the variety and three growing seasons (Table 1). It can be stated that tomatoes of all the varieties grown under HPSL have more dry matter (Table 1 and **Figure 5**).

**Table 1:** *P*-values (Kruskal-Wallis test) of the effects of different supplementary lightings on tomato fruit quality (*n* = 118).

**Parameter**	**“Bolzano”**	**“Chocomate”**	**“Encore”**	**“Diamont”**	**“Strabena”**
Fruit weight	0.013^*^	0.008^**^	0.110	0.400	0.560
Dry matter	0.022^*^	0.013^*^	0.011^*^	0.001^**^	0.015^*^
Soluble solids	0.027^*^	0.030	0.030^*^	0.001^**^	0.270
Acidity	0.078	0.022	0.160	0.001^**^	0.230
Taste index	0.370	0.140	0.600	0.001^**^	0.023^*^
Lycopene	0.052	0.290	0.860	0.160	0.920
β-carotene	<0.001^***^	0.007^**^	0.940	0.110	0.700
Phenols	0.097	0.750	0.450	0.800	0.420
Flavonoids	0.430	0.035^*^	0.720	0.440	0.170

Significance levels “^***^” 0.001, “^**^” 0.01, and “^*^” 0.05.

### Fresh Weight, Dry Matter, and Soluble Solids

**Figure 4:**
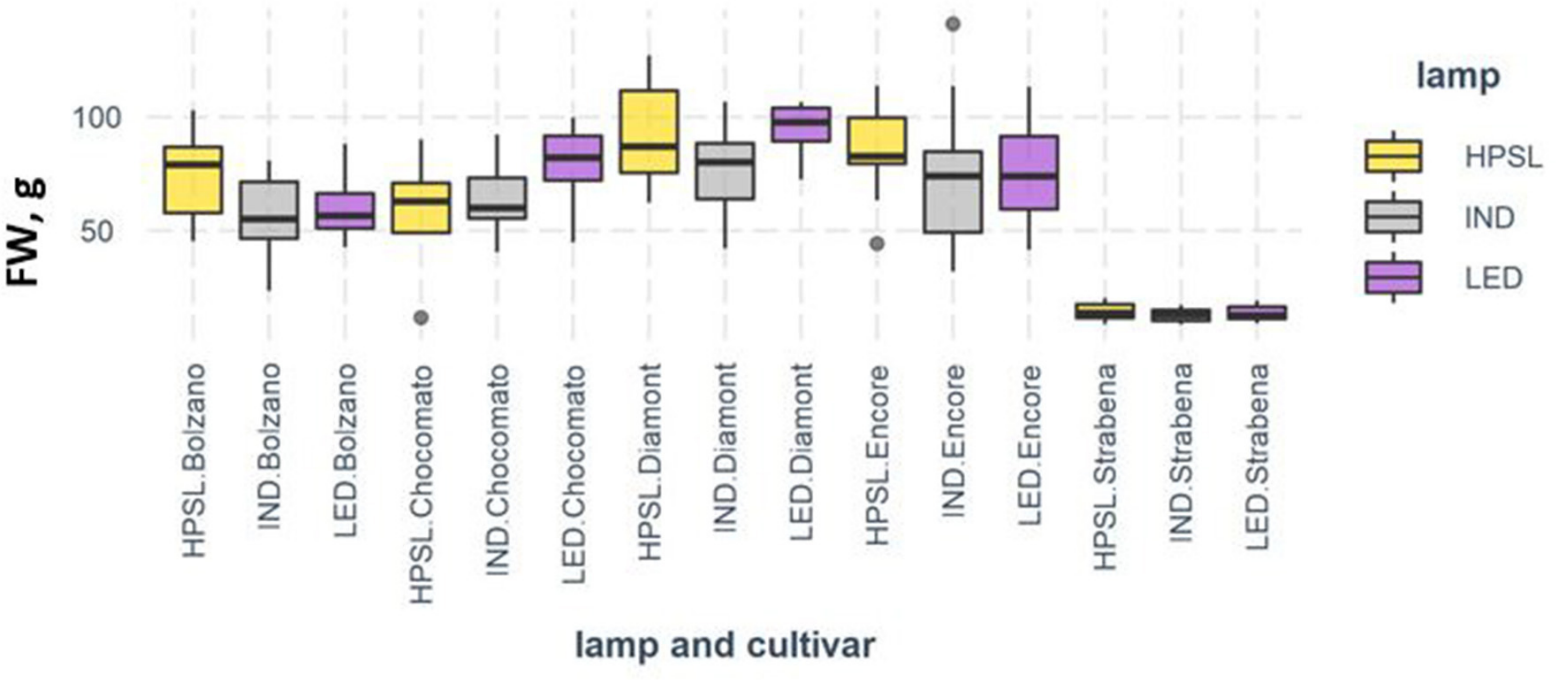
Fresh weight (g) of tomato fruits grown under different supplemental light sources.

The weight and size of the fruit depend significantly on the growing conditions of the plant. Although there were differences between the varieties, the average fruit of tomatoes growing under induction lamps was 12% smaller than under HPSL or LED. Different varieties seem to react differently to the supplementary LED light. Larger fruits are formed under the LEDs by “Chocomate” and “Diamont,” but the fresh weight of “Bolzano” is on average only 72% of the weight of tomato under HPSL. Fruits of “Encore” and “Strabena” grown under LED and IND supplementary lighting are similar in weight and are 10 and 7% smaller, respectively, than tomatoes grown under HPSL (Figure 4).

**Figure 5:**
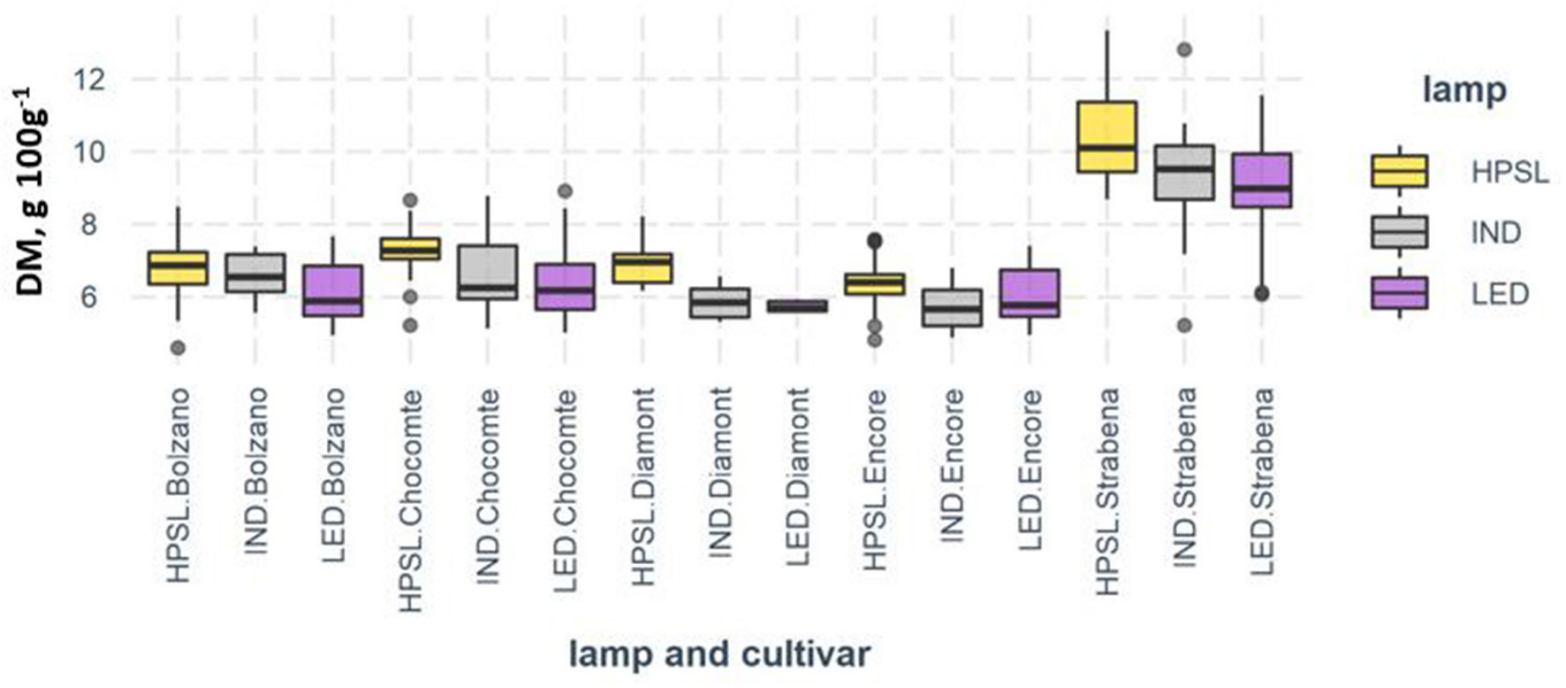
Dry matter (g 100 g^−1^) of tomato fruits grown under different supplemental light sources.

Dry matter content is one of the indicators of fruit quality. It correlates with the soluble solids content and influences tomatoes taste. In our experiments, the dry matter content of tomatoes varied between 46 and 113 mg g^−1^. The highest dry matter content (on average 95 mg g^−1^) was found for cherry variety “Strabena.” Among other tomatoes cultivars, the highest dry matter content (on average 66 mg g^−1^) was found in “Chocomate” (Figure 5).

During the experiment, the organic acid content, expressed as citric acid (CA) equivalent in tomatoes, averaged from 365 to 640 mg 100 g^−1^. The highest organic acid content was found in the cherry tomato cv “Strabena,” an average of 596 ± 201 mg CA 100 g^−1^, but the lowest organic acid content was found in the yellow fruit cv “Bolzano,” an average of 545 ± 145 mg CA 100 g^−1^. Organic acid content varied greatly not only between varieties, but also between sampling times; however, on average, higher organic acid content was found in tomatoes grown under IND lamps (exceeding HPSL and LED by 10.2%).

On average, the highest dry matter content was found in fruits grown under HPSL. Under the IND lamp, the dry matter content of tomato fruit decreases by 4.7–16.1%, below the LED of 9.9–18.2%. The varieties used in the experiments are differently sensitive to light. The smallest decrease in the dry matter under different light conditions was observed for cv “Strabena” (5.8% for IND and 11.1% for LED, respectively) and the largest decrease in the dry matter under different light conditions was observed for cv “Diamont” (16.1% and 18.2% respectively).

On average, soluble solids content varied between 3.8 and 10.2 °Brix. Similarly, for dry matter, the highest soluble solids content was detected in cherry tomatoes cultivar “Strabena” (on average 8.1 ± 1.0 °Brix). The tomato cv “Diamont” was the least sweet (on average 4.9 ± 0.4 °Brix).

**Figure 6:**
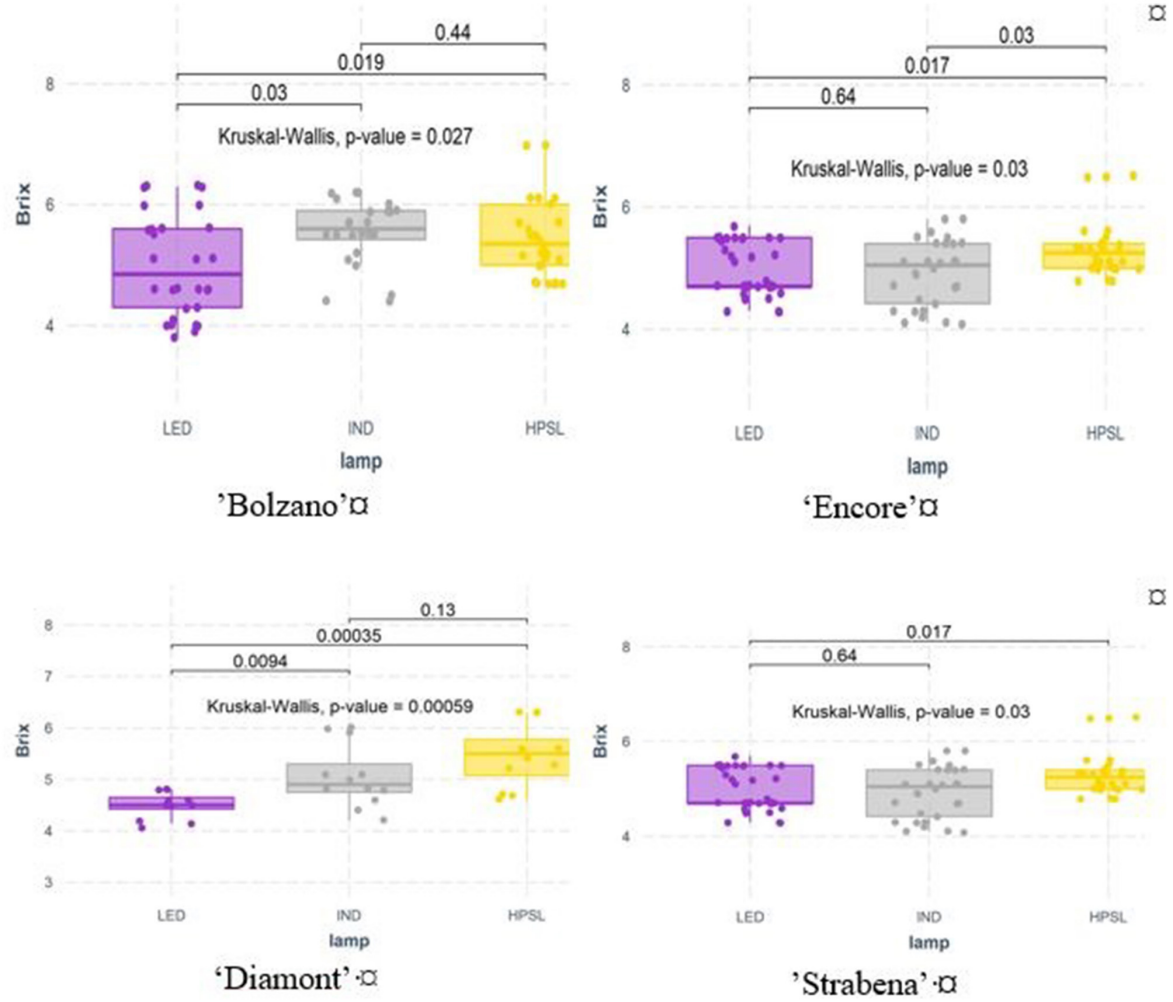
Soluble solids (Brix) in tomato fruits grown under different supplemental light sources.

Supplemental lighting significantly affected soluble solids content of tomato cultivars “Bolzano,” “Diamont,” and “Encore.” Under LED light, soluble solids content in these varieties significantly decreased in comparison with HPSL. The effect of the IND lamp was less. Under this lighting conditions, growing tomatoes of cv “Bolzano” and “Strabena” had on average 4.7 and 4.3% more sugar than under HPSL grown. Unfortunately, this increase is not statistically significant (Figure 6).

Tomatoes TI varies from 0.97 to 1.38. The tastiest was tomatoes of cv “Strabena,” on average TI was 1.32 ± 0.1 and the less tastiest was tomatoes of cv “Diamont,” on average TI was only 1.01 ± 0.06. High TI has tomato cultivar “Bolzano,” on average TI (1.12 ± 0.06), followed by “Chocomate,” on average TI (1.08 ± 0.06).

On average, the TI is not significantly affected by lighting source, except for cv “Strabena,” where the fruits under IND lamp have the TI increase in comparison with HPSL by 7.4% (LED by 4.2%) in comparison with HPSL and cv “Diamont” under both the previously mentioned lighting conditions decrease by 5.3 and 8.4%, respectively, was detected.

### Carotenoids Content

Lycopene concentration in tomatoes varied from 0.07 (cv “Bolzano”) to 7 mg 100 g^−1^ FM (“Strabena”). Slightly higher lycopene content in comparison with “Diamont” (4.40 ± 1.35 mg 100 g^−1^ FM) and “Encore” (4.23 ± 1.33 mg 100 g^−1^ FM) was found in brownish red-colored fruits of “Chocomate” (4.74 ± 1.48 mg 100 g^−1^ FM).

**Figure 7:**
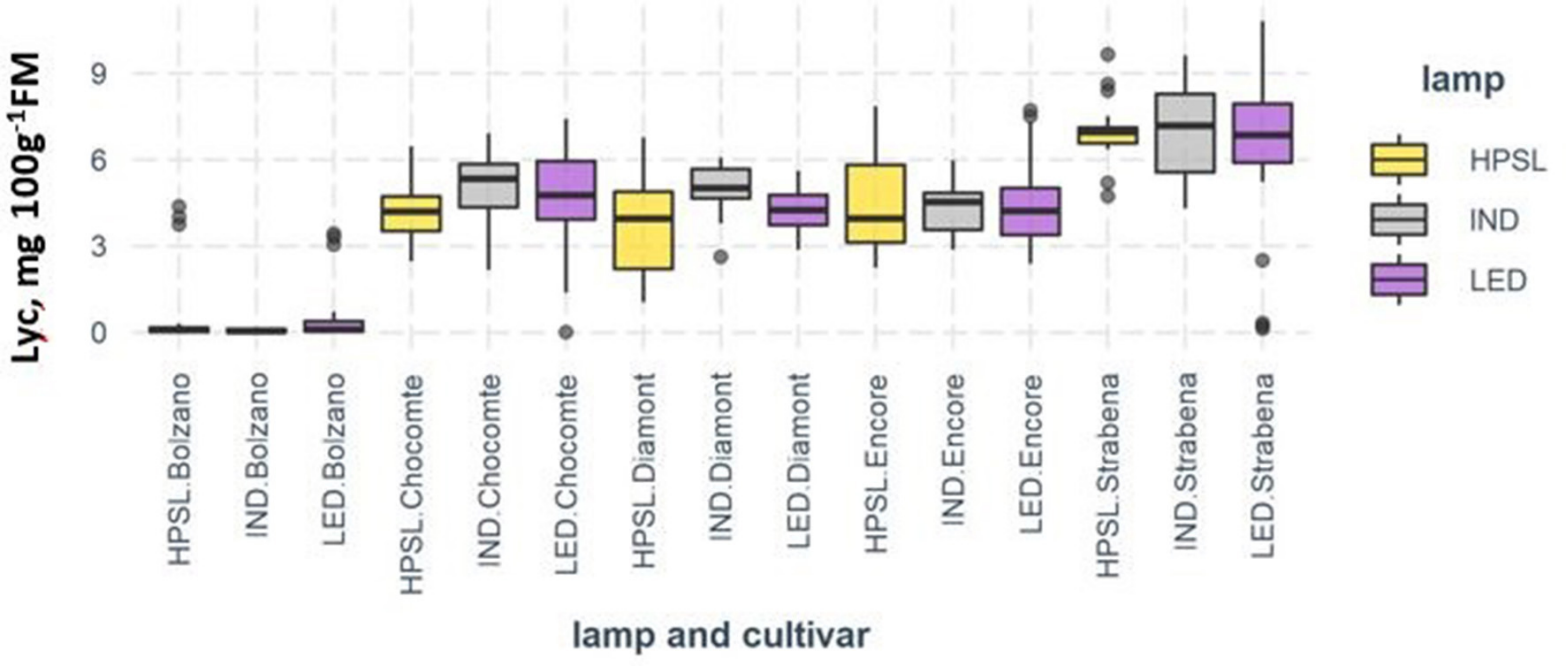
Lycopene content (mg 100 g^−1^ FM) in tomato fruits grown under different supplemental light sources.

On average, fruits from plants grown under IND lamps contain 17.9% more lycopene in comparison with HPSL. LED lighting has also promoted lycopene synthesis, but to a lesser extent, by an average of 6.5%. The effect of light sources has varied depending on the cultivar. The largest differences in lycopene biosynthesis were observed for “Chocomate.” The increase of lycopene content under IND compared to HPSL was 27.2% and below LED by 13.5%. “Strabena” was the least sensitive, with changes of 3.2 and −1.6%, respectively, compared to HPSL (Figure 7). Despite the relatively convincing results, the mathematical processing of the data does not confirm its reliability (Table 1).

During the experiment, β-carotene content in tomatoes averaged from 4.69 to 9.0 mg 100 g^−1^ FM. The highest β-carotene content was found in the cherry tomato cv “Strabena,” an average of 8.88 ± 1.58 mg 100 g^−1^ FM, but the lowest β-carotene content was found in the yellow fruit cv “Bolzano,” an average of 5.45 ± 1.45 mg 100 g^−1^ FM.

**Figure 8:**
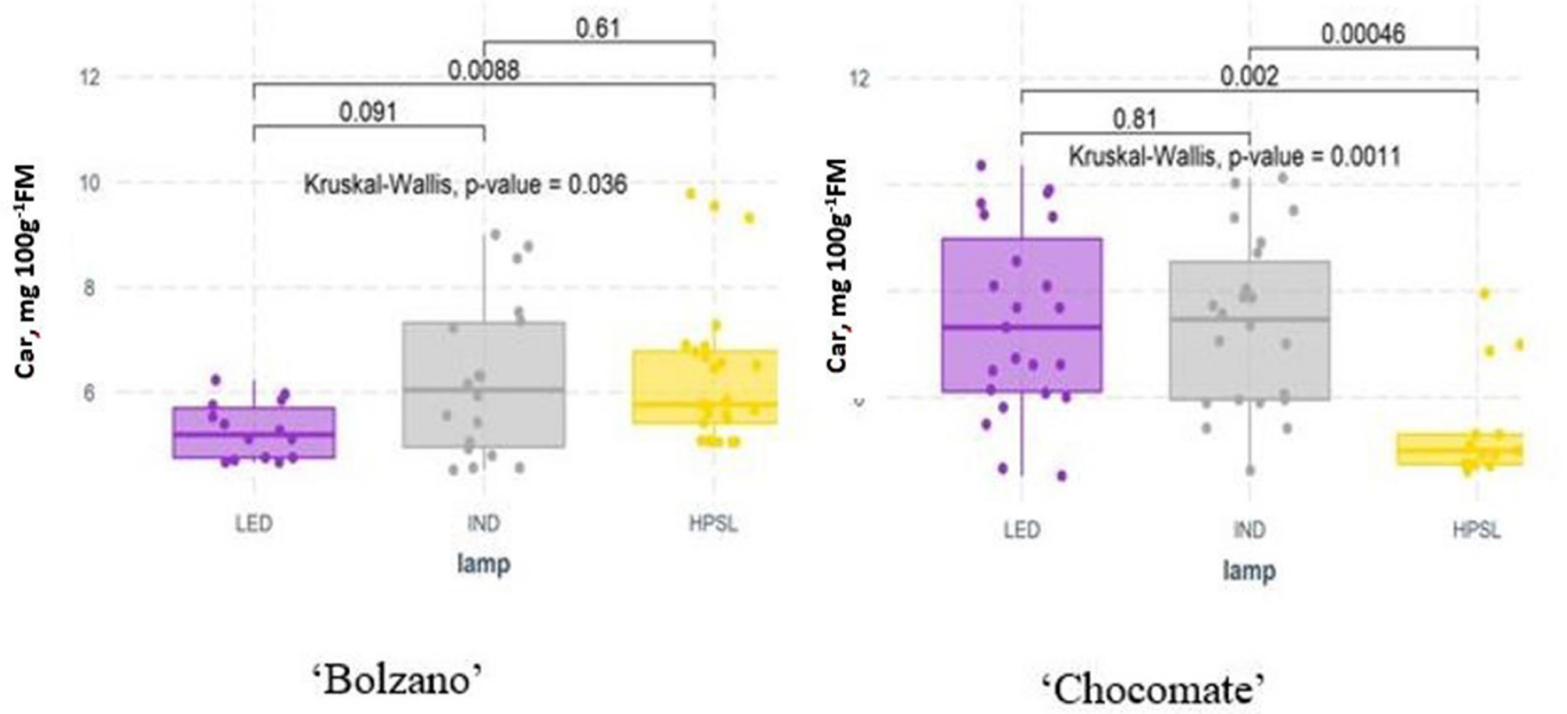
β-carotene content (mg 100 g^−1^ FM) in tomatoes cv ‘Bolzano' and ‘Chocomate' fruits grown under different supplemental light sources.

The significant differences in carotene content were found between varieties grown under different supplemental lighting. Cv “Bolzano” grown under LED shows a significant decrease in carotene content (by 18.5% compared to HPSL), while “Chocomate” has the lowest carotene content just below HPSL in tomato fruit (5.32 ± 1.08 mg 100 g FM^−1^) and it was increased by 34.3% under LED and 46.4 % under IND lamps (Figure 8).

### Total Phenolics and Flavonoids Content

The phenol content of tomato fruits varies on average from 27.64 to 56.26 mg GAE 100 g^−1^ FM (Table 2). The highest phenol content is observed for the variety “Strabena” and the lowest phenol content is observed for the variety “Diamont.” The phenol content of tomatoes varies according to the ripening season of the fruit, so there are large fluctuations between different sampling times. This leads to the fact that the differences between the tomatoes grown under different lamps are not significant.

**Table 2:** Content of total phenolics [mg gallic acid equivalent (GAE) 100 g^−1^ FM] and flavonoids [mg citric acid (CA) 100 g^−1^ FM] in the tomato fruits grown under different supplemental lighting.

**Parameter**	**“Bolzano”**	**“Chocomate”**	**“Encore”**	**“Diamont”**	**“Strabena”**
**Phenols**
HPSL	36.33 ± 5.34	31.23 ± 5.67	27.64 ± 7.12	30.26 ± 5.71	48.70 ± 11.24
IND	33.21 ± 4.05	34.77 ± 6.39	31.00 ± 6.02	30.63 ± 5.11	56.26 ± 13.59
LED	36.16 ± 6.41	31.70 ± 6.80	30.44 ± 3.01	30.98 ± 6.52	52.57 ± 10.41
**Flavonoids**
HPSL	4.50 ± 1.32	3.78 ± 0.65a	2.65 ± 1.04	2.57 ± 1.15	5.17 ± 2.33
IND	4.57 ± 0.75	5.24 ± 0.79b	4.96 ± 1.46	2.84 ± 0.67	6.65 ± 1.64
LED	4.96 ± 1.08	4.37 ± 1.18ab	3.02 ± 1.04	2.88 ± 1.08	5.91 ± 1.20

*Significantly different means are labeled with different letters*.

Although significant differences between the supplemental light variants appear only in the case of the cv “Chocomate,” the average flavonoid content of fruits grown under the lamp is by 33.3%, but below the LED by 13.3% higher. Under IND lamps, large differences between varieties are observed, but below LED the variability is in the range of 10.3–15.6%.

Experiments have shown that different tomato varieties react differently to the supplemental lighting used.

**Figure 9:**
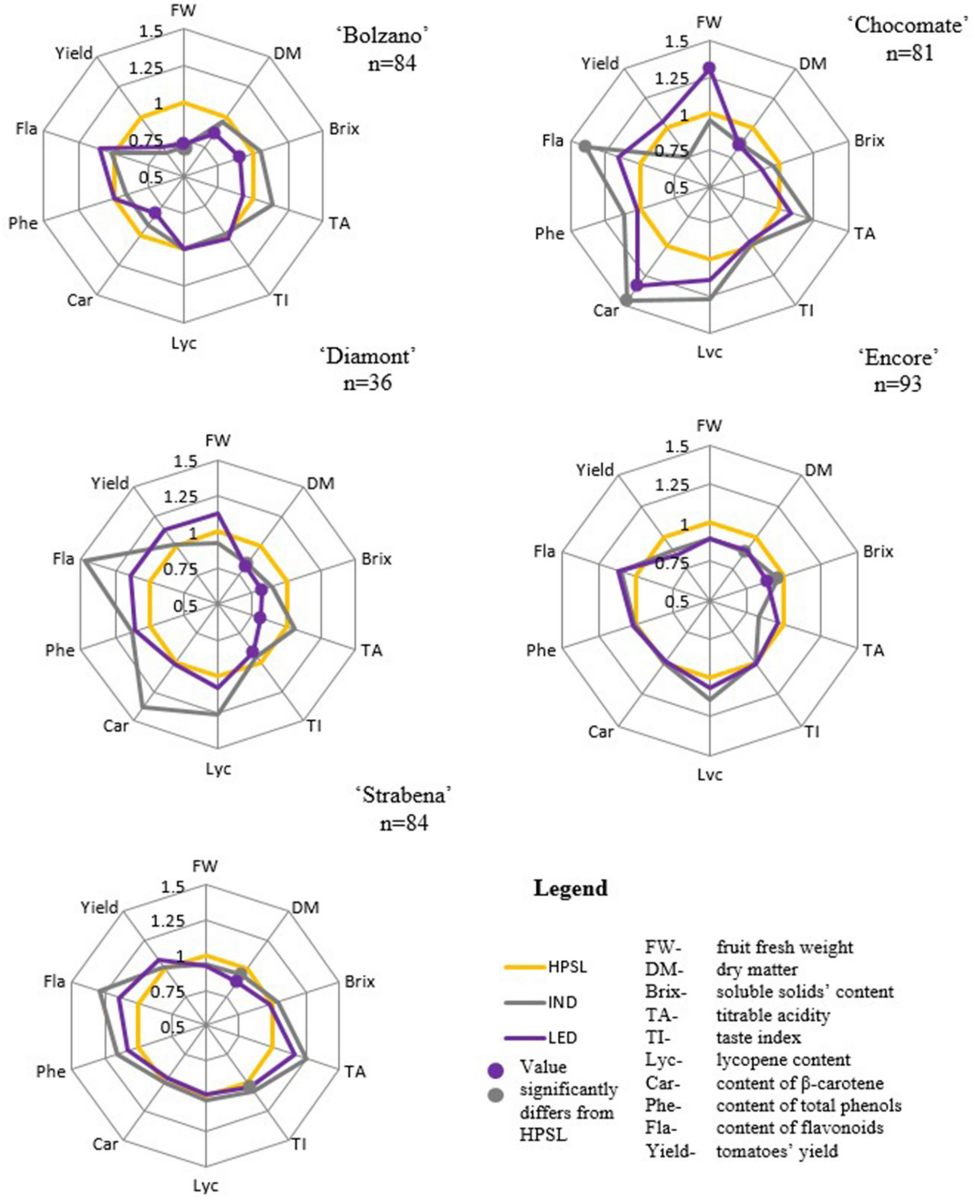
Ratio of the obtained parameters under supplemental light-emitting diode (LED) and induction (IND) lamps to high-pressure sodium lamp (HPSL).

It is not recommended to grow cv “Bolzano” under LED or IND lamp because in this lighting, the parameters are similar to those obtained under HPSL or significantly lower. Under LED lamps, the weight of one fruit, dry matter, soluble solids content, and carotene are significantly reduced (Figure 9).

Unlike “Bolzano,” “Chocomate” under LED lighting increases the weight of one fruit and the amount of carotene increases. Other parameters excluded dry matter and soluble solids content are also higher than in fruits obtained under HPSL. In the case of this variety, the induction lamp also shows good results (Figure 9).

For the cv “Diamont,” the indicators that determine the taste properties are significantly reduced under LED light, but the content of pigments and flavonoids is increased (Figure 9).

Cultivars “Encore” and “Strabena” are the most unresponsive to supplemental light treatment. For “Encore,” the only parameter significantly affected by the LED light spectrum is the soluble solids content. “Strabena” is also relatively tolerant on the changes in the spectral composition of light. This could be due to the genetic characteristics of the variety, as this was the only cherry tomato variety included in the experiment. It was characterized by significantly higher all the studied parameters. Therefore, it was not possible to detect changes in the studied parameters under the influence of light (Figure 9).

## Discussion

The average weight of the tomato fruit correlates with the intended weight of the variety; though, it is not achieved. This could be due to the cultivation method rather than the quality of the lighting, as less water can be used in a peat substrate, which may reduce the weight of the fruit, but increase the concentration of the active substances and improve the saturation of the taste (24). The smallest fluctuation of the average fruit weight of the “Encore F1” as a result of the lighting source could indicate a tolerance of this variety to quality of lighting. This corresponds with the review of the subject (25). The yield and quality of tomatoes are affected not only by the intensity of the supplemental light used, but also by its quality. Results show that lesser yield formed under IND lamps. However, it could be possible that lesser results showed due to smaller intensity of induction lamps in spite of the fact that main feature of induction lamps is broader green waves band. The data shows that the increase in the amount of red light contributes to the increase in the fresh weight of the tomatoes, but does not affect the increase in the dry matter content. It seems that the red light has stimulated the increase in the water content in the tomatoes. In contrast, the increase in blue light reduces the dry matter content of all tomato varieties. The least sensitive is yellow tomatoes cultivar “Balzano”. Several researches showed that photosynthesis under a combination of red and blue light tends to be higher than under HPS lighting, but fruit yield is equal (12). Olle and Virsile (26) found that red LEDs enhance tomatoes yield and that underlines findings of our research that states that generally with higher addition of red waves increases yield. In similar opinion, Zhang et al. (14) defines that even adding FR light in combination with red LEDs and HPSL increases total fruit number. Supplemental blue and red LED light resulted in the early ripening of tomato fruit. This could indicate that reason for higher fruit mass under LEDs for “Chocomate F1” and “Diamont F1” cultivars, since early ripening led to earlier setting of new fruits. In terms of yield, our data show that it is not the increase in red light that is more important in increasing yields, but the increased proportion of red light over blue light.

Since one of the beloved trait of tomato of the customer is sweetness, it is important to understand the possible ways of enhancing this feature. Nevertheless, it is usually altered by various environmental factors (27). There are evidences that the qualitative composition of light also affects the biochemical content of tomato fruit. Soluble sugar contents of the ripe tomato fruit were decreased by longer FR light durations (15). Kong et al. (16) results showed that blue light treatment significantly led to more total soluble solids. Sugar contents in plants are increased by green, blue and red light (28). Our experiments do not confirm that, because increasing of both blue and red light separately reduced the soluble solids content in most cases. Our results showed that the highest level of soluble sugars were found under HPSL which brings the largest proportion of red light than other lamps and also raises the temperature near the lamps. This correspondences with earlier researches where studies of Erdberga et al. (29) showed that content of soluble sugars, organic acids increase with increasing red waves doses. Similar results were obtained in other studies. A higher mean tomato fruit weight was obtained in plants supplementary lighted with HPS lamps as compared to plants from LED lamps (8.7–12.2% depending on cultivar) (30).

However, studies of Dzakovich et al. (31) proved that supplemental light quality (HPSL *via* LEDs) did not significantly affect the physicochemical (total soluble solids, titratable acidity, ascorbic acid content, pH, total phenolics, and prominent flavonoids and carotenoids) or sensory properties of greenhouse-grown tomatoes. This shows that the amount of soluble sugars in fruits can be affected not only by individual factors, but also by their combinations. Also in our experiments it was not possible to find regularities between influences of light on the acid content. In particular, future research should focus not only on the relationship between species and light, but also on the relationship between cultivar and light. Dry matter content was higher in “Chocomate F1” and “Strabena F1.” This corresponds with Kurina et al. (6), where on average, the red-brown accessions accumulated more dry matter (6.46%). Studies of Duma et al. (32) showed that when comparing fruits mass and TI, it is observed that higher TI is for smaller or bigger tomatoes. Experiments of Rodica et al. (23) showed that cherry and brownish red-colored tomatoes contain more soluble solids. In this study, it is underlined that quantity of the organic compounds determining the fruit taste depends on the yield of the cultivar.

The exposure to supplementary red and blue LED lighting increases the lycopene and β-carotene content (13, 29, 33, 34). Dannehl et al. (12) studies have shown that lycopene and lutein contents in tomatoes were 18 and 142% higher when they were exposed to the LED fixture. However, β-carotene content was not different between the light treatments. Ntagkas et al. (35) showed that zeaxanthin, the product of β-carotene conversion, increases in tomato fruits under blue and white light. In this study, these statements partly are true only in case of “Bolzano F1” where significantly larger amount of lycopene were found under LED treatment, but β-carotene did respond negatively to this treatment. This could be due to genetic features since “Bolzano F1” is only orange-fruited cultivar in this study. In other studies, with red-fruited and brown cultivars, highest amount of lycopene and β-carotene were found under Induction lamps which do not confirm the trends of previous years (29). Our experiments showed that the lycopene content of all red fruit tomato cultivars increased with increasing of blue light. In contrast, changes in carotene content in different cultivars fail to establish regularities common to all tomato cultivars used in the experiments. This discrepancy points to the need for additional testing of subject in the future. Same pattern of response to light due to cultivar features was observed with amount of phenols and flavonoids. All the red-fruited and brown-fruited cultivars showed better results under IND lamps, while “Bolzano F1” responded with higher results to HPSL and LED lamps with no significant difference. This study corresponds with the findings of Kong: the blue light treatment significantly led to more concentration of individual phenolic compounds (chlorogenic acid, caffeic acid, and rutin) (16). Continuous red light significantly increased lycopene, β-carotene, total phenolic content, total flavonoid concentration, and antioxidant activity in tomatoes (36). In our earlier studies, flavonoids changed fluctuating; therefore, no effects of light wavelength should be noted as significant.

The amount of phenols increased with the growing proportion of blue light provided by LED lamps (29), this corresponds also with our research. It is mentioned in other researchers' works that exposure to either UV or LED light had no effect on total phenolic compounds, despite the fact that both the light treatments are known to modulate the expression of an array of genes involved in the biosynthesis of phenolic compounds and carotenoids (36). There should be mentioned that similarly with the weight of the fruit, there are no significant differences in chemical compounds in “Encore F1” due to light treatment. This allows to declare that cultivar “Encore F1” could be tolerant to composition of light. Our experiments confirm the literature data that the synthesis of secondary metabolites is enhanced by both the quantitative amount of blue light and the increased proportion of blue light in the overall lighting system.

The results obtained show that the chemical components, including the acid-soluble sugars and their ratio, which are responsible for the characteristic taste of the variety, depend primarily on the genetics of the variety. The good taste of tomatoes is characterized not only by the combination of species-specific pigments and biologically active substances, but also by their amount. In particular, the ratio and quantity of acids and sugars characterize the saturated and high-quality taste. In this study, the positive correlation between soluble sugars and titratable acids is ~0.4, which is correlated with research of Hernández Suárez, where the positive correlation between the two indicators was found to be 0.39 (37). In studies of Dzakovich et al. (31), tomatoes were profiled for total soluble solids, titratable acidity, ascorbic acid content, pH, total phenolics, and prominent flavonoids and carotenoids. Their studies indicated that greenhouse tomato fruit quality was only marginally affected by supplemental light treatments. Moreover, consumer sensory panel data indicated that tomatoes grown under different lighting treatments were comparable across the lighting treatments tested. Study suggested that the dynamic light environment inherent to greenhouse production systems may nullify the effects of wavelengths of light used in their studies on specific aspects of fruit secondary metabolism (31). This is partly in line with this study, as the figures obtained do not show clear and unambiguous trends, which allow us to say that one of the lighting is more useful for tomatoes than the others. However, certain lamps may be used for certain varieties, for example, HPSL lamps would be more suitable for “Bolzano F1” and LED lighting is recommended for “Chocomate F1.” This corresponds with study were effect of different geographical latitudes on the chemical properties of tomatoes was studied. Bhandari et al. (38) clarified that while the combination of the position of the sun toward the sky and, consequently, the combination of visible light waves, it plays an important role in changing the chemical composition of tomatoes; there are varieties that are immune to these processes. All these conclusions allow to underline that chemical composition of tomato is primarily dependent on genotype, since cultivars relationships with growing factors, particularly with lighting, are genetically predisposed.

## Conclusion

Different tomato varieties react differently to the supplemental lighting used. Cultivars “Encore” and “Strabena” are the most unresponsive to supplemental light. For “Encore,” the only parameter significantly affected by the LED light spectrum is the soluble solids content. “Strabena” is also relatively tolerant on the changes in the spectral composition of light. This could be due to the genetic characteristics of the variety, as this was the only cherry tomato variety included in the experiment. It is not recommended to grow orange color fruit cv “Bolzano” under LED or IND lamp because in this lighting, the parameters are at the level of HPSL or significantly worse. Under LED lamps, the weight of one fruit, dry matter, soluble solids content, and β-carotene are significantly reduced. The one fruit weight and the amount of β-carotene of red-brown color fruit cv “Chocomate” under LED lighting significantly increases. Other parameters excluded dry matter and soluble solids content are also higher than in fruits obtained under HPSL.

Experiments have shown that HPSL stimulates the accumulation of primary metabolites in tomato fruit. In all the cases, soluble solids content was 4.7–18.2% higher as compared to other lighting sources.

As LED and IND lamps emit about 20% blue-violet light, the results suggest that this part of the spectrum stimulates the accumulation of phenolic compounds in the fruit by 1.6–47.4% compared to HPSL. The content of carotenoids as secondary metabolites depends on both the variety and the light source. Red fruit varieties tend to synthesize more β-carotene under supplemental LED and IND light.

The blue part of the spectrum plays a greater role in ensuring crop quality. An increase or quantification of its proportion in the total spectrum promotes the synthesis of secondary metabolites (lycopene, phenols and flavonoids), leading to a decrease in dry matter and soluble solids content.

Given the large effect of genotypic variability in the tomatoes and light relations, further study should continue to focus on the combinations of cultivars and different supplemental light spectra to increase the content of biologically active compounds.

## Data Availability Statement

The raw data supporting the conclusions of this article will be made available by the authors, without undue reservation.

## Author Contributions

IE was incharge of tomatoes cultivation and sampling, laboratory work, compounds quantification, and also contributed to the writing of the manuscript. IA brought up the idea, contributed to the study conception and design, was incharge of tomatoes sampling, laboratory work, compounds quantification, and also contributed to the writing of the manuscript. MD contributed to the study conception and design, optimization of analytical methods, analyzed the samples in the laboratory, and made recommendations and suggestions. RA contributed to the statistical analysis, interpretation of data, and made recommendations and suggestions regarding the manuscript. LD contributed to the study conception and design, was incharge of tomatoes sampling, laboratory work, compounds quantification, and made recommendations and suggestions regarding the manuscript. All authors contributed to the article and approved the submitted version of the manuscript.

## Funding

This study was funded by the Latvian Rural Development Program 2014-2020 Cooperation, call 16.1 project Nr. 19-00-A01612-000010 Investigation of innovative solutions and new method development for efficiency and quality increase in Latvian greenhouse sector (IRIS).

## Conflict of Interest

The authors declare that the research was conducted in the absence of any commercial or financial relationships that could be construed as a potential conflict of interest.

## Publisher's Note

All claims expressed in this article are solely those of the authors and do not necessarily represent those of their affiliated organizations, or those of the publisher, the editors and the reviewers. Any product that may be evaluated in this article, or claim that may be made by its manufacturer, is not guaranteed or endorsed by the publisher.
